# Dual-parameter tomographic imaging of attenuation and backscattering coefficients for quantitative evaluation of immune cell-mediated cytotoxicity in tumor spheroids

**DOI:** 10.7150/thno.118722

**Published:** 2025-08-22

**Authors:** Seokgyu Han, Ingyoung Kim, Baekcheon Seong, Woovin Kim, Hongseong Kim, Sein Kim, Chulmin Joo, Sungsu Park

**Affiliations:** 1 School of Mechanical Engineering, Sungkyunkwan University, Suwon, 16419, Republic of Korea.; 2 Department of Mechanical Engineering, Yonsei University, Seoul, 03722, Republic of Korea.; 3 Department of Biomedical Engineering, Sungkyunkwan University, Suwon, 16419, Republic of Korea.; 4 Institute of Quantum Biophysics (IQB), Sungkyunkwan University, Suwon, 16419, Republic of Korea.; 5 Department of Metabiohealth, Sungkyunkwan University, Suwon, 16419, Republic of Korea.

**Keywords:** tumor spheroid, chimeric antigen receptor T cell, optical coherence tomography, attenuation, backscattering

## Abstract

**Rationale:** Quantitative, non-perturbative assessment of immune cell-mediated cytotoxicity in tumor spheroids remains challenging due to the lack of real-time label-free analytical tools. Conventional methods such as fluorescence imaging or biochemical assays often require labeling and provide limited longitudinal analysis, which prohibits dynamic monitoring of therapeutic responses. This study presents a dual-parameter tomographic analysis method that simultaneously quantifies attenuation coefficient (AC) and backscattering coefficient (BSC) from optical coherence tomography (OCT) datasets, enabling dynamic evaluation of therapeutic responses in three-dimensional (3D) tumor spheroids.

**Methods:** We developed a 3D Gabor transform-based algorithm to extract depth-resolved AC and BSC metrics from OCT volumetric datasets. Unlike conventional strategies, our method enables simultaneous voxel-wise measurements of AC and BSC values, with superior noise robustness. Experiments with intralipid solutions across a range of concentrations revealed that the Gabor-based approach yielded AC and BSC estimations with more than three times greater precision than prior methods. This approach enables high-resolution measurements of structural and optical property changes associated with apoptosis, allowing spatial and temporal mapping of treatment-induced cytotoxicity in HER2-positive breast tumor spheroids treated with AZD4547 and HER2-targeted chimeric antigen receptor (CAR) T cells.

**Results:** In AZD4547-treated spheroids, AC increased dose-dependently from 0.39 to 0.64, reflecting a 64% rise, while BSC increased from 0.09 to 0.12, an approximate 33% increase. CAR T cell treatment induced a rapid, spatially progressive increase in both AC and BSC, originating at the spheroid periphery and advancing inward. Over 12 hours, AC rose from 0.40 to 0.82 (2-fold increase) and BSC from 0.09 to 0.20 (2.2-fold increase). While AC and BSC individually correlated with spheroid viability, their combined analysis consistently achieved a higher coefficient of determination (R² = 0.98) across both treatment modalities.

**Conclusions:** This dual-parameter OCT-based assay framework provides a sensitive, label-free method for distinguishing between immune- and drug-induced apoptosis in tumor spheroids. Its strong correlation with viability and capacity to resolve spatially resolved dynamics underscore its potential for robust, *in situ* assessment of immunotherapeutic efficacy.

## Introduction

Chimeric antigen receptor (CAR) T cells have overcome many limitations of traditional cancer therapies by enhancing the ability of T cells to specifically recognize and eliminate cancer cells, particularly in hematologic malignancies such as leukemia and lymphoma 1. Despite these successes, the efficacy of CAR T cell therapy in solid tumors remains unproven. This limitation is largely attributed to the tumor microenvironment (TME), which presents several barriers, including physical constraints, immunosuppressive factors, and heterogeneous antigen expression, all of which impede CAR T cell function 2, 3. The migration of cytotoxic T lymphocytes is particularly hindered by the dense tumor matrix and abnormal vasculature, restricting T cell infiltration and movement within tumor sites 4-6.

Furthermore, evaluating the efficacy of CAR T cells in solid tumors is challenging owing to the high cost and limited physiological relevance of animal models, which often fail to accurately replicate the human TME, especially in terms of immune response and tumor biology. These discrepancies can lead to an overestimation of CAR T cell effectiveness in preclinical studies 7. In response, significant efforts have been directed toward developing advanced *in vitro* models, such as tumor spheroids, which better recapitulate human tumor conditions and offer a more reliable platform for evaluating CAR T cell therapies. These approaches aim to extend the benefits of CAR T cell therapy to a broader range of cancers, including solid tumors 8.

Tumor spheroids, which are three-dimensional (3D) aggregates of cancer cells, have been widely employed to model the TME of solid tumors 9. They are frequently used to evaluate the efficacy of therapeutic drugs and cytotoxic immune cells 10. These models mimic the dense cellular architecture that impede immune cell infiltration and reproduce the hypoxic core that drives immunosuppressive signaling 11. However, current methods for assessing therapeutic efficacy in spheroids often involve disruptive and labor-intensive steps such as washing and separation, making them unsuitable for real-time or longitudinal analysis. For instance, assays like adenosine triphosphate chemiluminescence 12 and lactate dehydrogenase cytotoxicity 13 require spheroid disruption. While fluorescence-based assays avoid physical disruption, they still necessitate complex sample preparation, including staining and washing 14, 15. More advanced techniques, such as fluorescence-activated cell sorting, require separation of cancer and immune cells, adding further complexity 8. Moreover, most of these techniques yield only endpoint measurements, limiting their utility for dynamic monitoring.

To address these limitations, imaging-based, non-destructive evaluation methods are increasingly being explored. Among various 3D imaging modalities 16-19, optical coherence tomography (OCT) stands out for its ability to provide high-resolution, label-free, 3D imaging of turbid biological tissues at depths of several millimeters 20. Recent studies have demonstrated the potential of OCT in monitoring volumetric growth and drug responses in spheroid models 21-24. In addition, numerous computational algorithms have been developed to extract structural and functional information from OCT datasets, enabling differentiation of necrotic regions and analysis of dynamic responses. For instance, the optical attenuation coefficient (AC) has been used to delineate necrotic zones, while dynamic OCT metrics, such as mean frequency, logarithmic intensity variance (LIV), and correlation decay speed, have been applied to quantify intracellular motility 23, 24.

The optical AC is influenced by the density and refractive index (RI) contrast of scatterers and absorbers within the tissue. Higher scatterer density and greater RI mismatch, along with increased absorption, typically result in high AC values. Traditional AC estimation relies on single-exponential decay models that assume homogenous media, which are inadequate for resolving depth-dependent variations caused by tissue heterogeneity and layered structures 25, 26. To overcome these limitations, advanced methods such as frequency-domain 27 and overestimation-free methods 26 have been proposed to mitigate artifacts arising from incomplete signal decay and noise-floor interference in complex tissues 27, 28.

Backscattering coefficient (BSC), which quantifies the fraction of light redirected toward the detector, is influenced by particle size, shape, RI contrast, and tissue composition, offering complementary information to AC (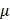). According to scattering theory, the BSC (*β*) is inversely proportional to the particle size (r) for particles larger than the illumination wavelength. This relationship has been effectively utilized to differentiate various tissue types such as lipid-rich, fibrous, and calcified plaques 29 and to characterize layered tissue architectures 30. For example, Xu *et al.* 29 demonstrated that fibrous plaques, composed of densely packed small fibers, exhibit high BSC in OCT images. In contrast, lipid-rich and calcified plaques, which contain particles comparable to or larger than the illumination wavelength, predominantly scatter light forward, resulting in lower BSC. These BSC variations, driven by differences in particle size, shape, and RI mismatch, form the basis of key contrast mechanisms in intravascular OCT imaging.

**Figure 1:**
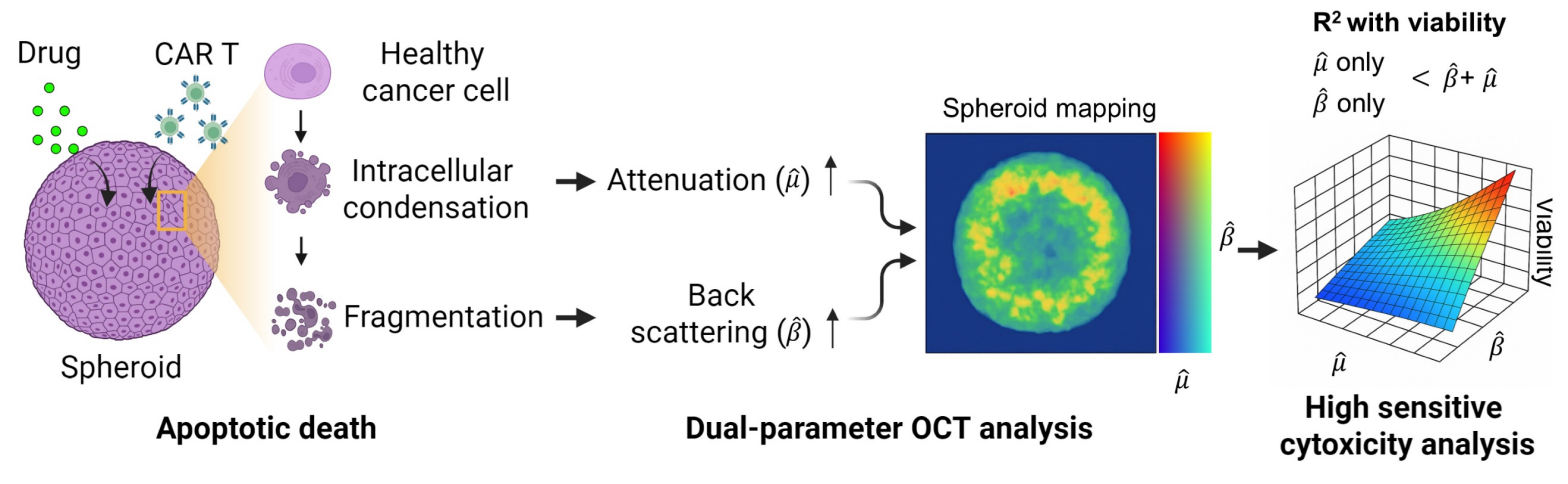
Dual-parameter OCT analysis for cytotoxicity assessment. Apoptotic changes in tumor spheroids increase normalized optical AC (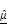) and BSC (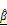), which are captured by OCT. Combined 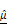
and 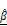
analysis enables highly sensitive, label-free evaluation of cytotoxic responses with high correlation to viability.

Tumor spheroids, as packed 3D assemblies, undergo rapid nano- to microscale structural remodeling when challenged with pharmacologic agents or cytotoxic immune cells; therefore, capturing these structural and optical property changes requires spatially resolved, noise robust quantification of scattering characteristics such as AC and BSC. Here, we introduce a novel OCT-based tomographic analysis that simultaneously maps AC and BSC with high resolution, enabling quantitative, non-destructive, and real-time monitoring of CAR T-cell-mediated cytotoxicity in tumor spheroids (**Figure 1**). Our implementation employs a complex-valued 3D Gabor filter, which enables high-precision AC and BSC estimation with markedly higher noise-robustness than conventional log-and-fitting (LF) 31 or Fourier-domain (FD) methods 27 (**Table S1, Figure S1**). To our knowledge, this is the first application of combined AC and BSC metrics to the quantitative analysis of tumor spheroids 29, 32-36. In our experiments with intralipid solutions, our method demonstrated approximately 23-fold and 4-fold improvements in AC detection accuracy compared to LF 31 and FD techniques 27, respectively. Moreover, our BSC measurement featured a high linearity with the intralipid concentrations (R^2^ = 0.99) and a coefficient of variation (CV) of < 1.84% (**Table S1, Figure S1**).

We applied our method to the analysis of human epidermal growth factor receptor 2 (HER2)-positive breast tumor cell (BT474) spheroids treated with the fibroblast growth factor receptor (FGFR) inhibitor AZD4547 37. Linear regression analysis was performed to assess the relationship between AC, BSC, and spheroid viability in drug-treated samples, both individually and in combination. Subsequently, the method was explored to evaluate the cytotoxic response of tumor spheroids to HER2-targeted CAR T cells, evaluating structural changes along with alterations in AC and BSC. This approach enabled the visualization of spatially distinct patterns of CAR T cell-induced cytotoxicity, including signal features consistent with apoptotic activity. Our findings demonstrate the potential of AC and BSC tomographic imaging as powerful, label-free tools for the quantitative evaluation of CAR T cell efficacy, offering a promising platform for accelerating the development of effective immunotherapies.

## Materials and Methods

### Materials

Methyl cellulose (MC), Triton X-100, normal donkey serum (NDS), Alexa Fluor 488-conjugated goat anti-rabbit IgG secondary antibody, 4',6-diamidino-2-phenylindole (DAPI) and propidium iodide (PI) were purchased from Sigma-Aldrich (St. Louis, MO, USA). Minimum Essential Medium (MEM), MEM/F12, penicillin, and streptomycin were purchased from Life Technologies (Carlsbad, CA, USA). Dulbecco's Modified Eagle medium, Roswell Park Memorial Institute (RPMI)-1640 medium, and fetal bovine serum (FBS) were purchased from HyClone Laboratories (Logan, UT, USA). Polydimethylsiloxane (PDMS) was purchased from Dow Corning Co. (Midland, MI, USA). The LIVE/DEAD™ Viability/Cytotoxicity Kit was purchased from Molecular Probes (Eugene, OR, USA). The FGRF inhibitor AZD4547 was purchased from Selleck Chemicals (Houston, TX, USA) and EZ-Cytox was purchased from Daeillab Service (Seoul, Korea). MitoTracker™ was purchased from Thermo Fisher Scientific (Waltham, MA). 4% paraformaldehyde (PFA) was purchased from Tech & Innovation (Seongnam, Korea). Anti-Calnexin primary antibody was purchased from Abcam (Cambridge, UK).

### Cell Culture

The HER2-positive breast cancer cell line BT474, originally derived from a female patient, was obtained from the American Type Culture Collection (ATCC, Bethesda, MD, USA). The cell line was authenticated by ATCC using short tandem repeat (STR) profiling and confirmed to be free of mycoplasma contamination. BT474 cells were cultured in RPMI-1640 medium supplemented with 10% (v/v) FBS, 100 U/mL penicillin, and 100 μg/mL streptomycin.

The generation of mock and HER2 CAR vectors, as well as the development of CAR T cells, followed the protocol described in our previous publication on HER2 CAR T cell production 38. Briefly, the HER2 CAR retrovirus was produced using Phoenix-GP and PG13 packaging cells, while the mock virus was generated using parental PG13 cells. Human T cells were activated with anti-CD3/CD28 antibodies and transduced on RetroNectin-coated plates with either HER2 CAR or mock viral supernatants. Transduced T cells were expanded in the presence of interleukin-2 (IL-2) and cryopreserved until use.

Prior to experiments, HER2 CAR and mock T cells were thawed and incubated overnight in RPMI-1640 medium supplemented with 10% (v/v) FBS, 100 U/mL penicillin, and 100 μg/mL streptomycin. All cells were maintained at 37 °C in a humidified atmosphere with 5% CO₂.

### Spheroid Formation

For spheroid formation, 96-well plates with glass bottoms (ibidi, Gräfelfing, Germany) were coated with 50 µL of PDMS mixed with its curing agent at a 10:1 ratio and degassed in a vacuum chamber for 30 min 39. The plates were incubated at 80 °C for 2 h. The plates were cooled down at 25 °C in a biosafety cabinet. Following this, 100 μL of BT474 cell suspension in RPMI-1640 medium supplemented with 1% (v/v) MC was added to each well, with cell numbers ranging from 500 to 4,000. The plates were then incubated at 37 °C with 5% CO_2_ for two days to allow spheroid formation. Spheroids in a 96-well plate were randomly assigned to either the control or treatment group using simple randomization.

### Drug Treatment on Tumor Spheroids

Once single spheroids had formed in each of the plate wells, they were treated with different concentrations (0-100 μM) of AZD4547 diluted in RPMI-1640 medium. Subsequently, the cells were incubated at 37 °C with 5% CO_2_ for three days.

### LIVE/DEAD Staining and Viability Measurement

After drug treatment, 10 μL of LIVE/DEAD reagent—containing 20 μM calcein AM and 40 μM ethidium homodimer-1 (EthD-1)—was added to each well and incubated at 37 °C in a 5% CO₂ atmosphere for 30 min. Bright-field and fluorescence images of the spheroids were captured using a K1-Fluo confocal microscope (Nanoscope Systems, Daejeon, Korea). These images were subsequently analyzed using ImageJ software (NIH, Bethesda, MD, USA). Cell viability was assessed based on the relative intensities of green fluorescence (live cells) and red fluorescence (dead cells).

To determine the IC₅₀ value of BT474 spheroids treated with AZD4547, 10 μL of EZ-Cytox reagent, which contains water-soluble tetrazolium salts (WSTs), was added to each well and incubated for 1 h. The absorbance of the resulting WST-formazan product, formed by mitochondrial dehydrogenase activity, was measured at 450 nm using a microplate reader (Molecular Devices, San Jose, CA, USA). Cell viability was calculated by comparing the number of viable cells at various drug concentrations to the number of viable cells in untreated control cultures.

### Cytotoxicity Assay of HER-2 CAR T Cells

The cytotoxicity assay for HER2 CAR T cells was conducted similarly to the drug testing procedure. Spheroids were seeded at 500 cells per well, and 10 μL of RPMI-1640 medium containing either 2,000 or 8,000 mock or HER2 CAR T cells were added to each well. The plates were then incubated at 37 °C in a 5% CO₂ atmosphere for 12 h. LIVE/DEAD staining, image acquisition, and image analysis were performed as described in Section 2.5.

To monitor apoptosis induced by CAR T cells, green fluorescence labeled mock or CAR T cells were added to each well along with medium containing 100 μM PI, as previously reported 40. The plate was placed in a live-cell imaging chamber maintained at 37 °C with 5% CO₂, and fluorescence images were captured every 10 min for 15 h using a THUNDER imager (Leica, Wetzlar, Germany).

### Immunofluorescence

Mitochondrial and endoplasmic reticulum staining was performed on BT474 cells cultured as a monolayer in µ-Plate 96 Well. For mitochondrial labeling, cells were incubated with 250 nM MitoTracker diluted in complete culture medium for 15 min at 37 °C in a humidified CO₂ incubator. After incubation, the staining medium was removed, and cells were washed three times with PBS. Cells were then fixed with 4% PFA in PBS for 10 min at room temperature (RT) on a shaker, followed by three washes with PBS. Permeabilization was carried out using 0.1% Triton X-100 in PBS for min at RT with gentle shaking. After washing three times with PBS, cells were blocked with 10% NDS in PBS for 30 min at RT. Then, Endoplasmic reticulum (ER) staining was performed using an anti-Calnexin primary antibody diluted 1:500 in 10% NDS, and cells were incubated overnight at 4 °C on a shaker. After primary antibody incubation, cells were washed three times with 10% NDS in PBS, followed by a 1 h incubation at RT with Alexa Fluor 488-conjugated goat anti-rabbit IgG secondary antibody diluted 1:500 in 10% NDS. The samples were then washed three times with PBS. For nuclear staining, cells were incubated with DAPI (1:1000 in PBS) for 5 min at RT, followed by three additional PBS washes. Confocal fluorescence imaging was performed using the K1-Fluo confocal microscope.

### OCT System

We performed tomographic AC and BSC imaging of the tumor spheroids using a custom-built OCT system (**Figure S2**). The OCT setup employed a superluminescent diode (SLD) as the light source, with a center wavelength of 850 nm and a spectral bandwidth of 100 nm (M-T-850-HP-I, SUPERLUM, Carrigtwohill, Ireland). Light from the SLD was coupled into a broadband 50:50 fiber coupler (TW850R5A2, Thorlabs, USA) and split into reference and sample arms. In the sample arm, the light was collimated and deflected by a two-dimensional (2D) galvanometric scanner (GVSM002-EC, Thorlabs), and then focused onto the specimen using a microscope objective (PLN 4×/0.1, Olympus, USA). To achieve a large depth of focus (DoF, ~450 μm), we underfilled the back aperture of the objective lens using a beam with a diameter of 2 mm. The backscattered light from the specimen was combined with the reference beam in a fiber-based interferometer, and the resulting interference spectrum was recorded using a spectrometer (Cobra-S 800, Wasatch Photonics, USA) operating at an A-line rate of up to 50 kHz. The system achieved lateral and axial resolutions of approximately 10.1 μm and 3.3 μm, respectively. Upon the acquisition, the interference spectra were zero-padded by a factor of four, increasing the number of sampling points in the A-line intensity profile to 4,096. The OCT imaging volume encompassed 204 × 327 × 2,480 μm^3^ (x × y × z), with a voxel resolution of 500 × 500 × 4,096. To minimize the effect of the focusing beam profile on AC and BSC estimations, A-line measurements from the spheroids were normalized to those obtained from an intralipid solution. A Gabor transform-based analysis was then applied to generate spatially resolved AC and BSC maps across the OCT volume.

### Depth-resolved AC and BSC Measurement

The irradiance of an OCT beam traversing a homogeneous medium follows Lambert-Beer's law, which is mathematically expressed as 30, 41:


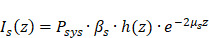

(1)

where 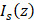
denotes the intensity measurement of the OCT beam after propagation through a medium over a distance 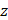, and 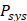
represents the system-related parameters 30. 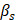
and 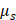
are the BSC and AC of the sample, respectively, and 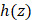
describes the point spread function (PSF) of the OCT instrument.

To achieve depth-resolved AC and BSC measurements, we first divided the A-line intensity measurement of the specimen (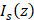) by that of a reference sample (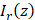) with AC and BSC values of 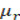
and 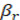, respectively. This division is expressed as


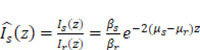

(2)

and mitigates errors caused by the PSF of the imaging system. In our experiments, a 1% intralipid solution served as a reference. The AC of this solution (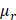) was independently measured using a spectrophotometer (V-650, JASCO, USA) and determined to be approximately 1.0 mm^-1^ at 850 nm (**Figure S3**). The BSC of the solution, however, was not provided by the spectrophotometer.

For a 3D OCT dataset, we perform this division for each A-line and apply a three-dimensional (3D) Gabor filter, defined as:


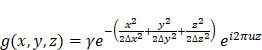

(3)

to achieve a spatially resolved AC and BSC mapping. Here, 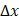, 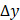, and 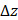
denote the 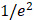
radii of Gaussian functions in the 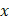, 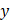, and 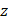
dimensions, respectively, and 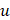
represents the spatial frequency along the z direction. Note that the Gabor filter in Eq. (3) was normalized by setting 
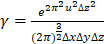
to ensure that 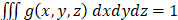. The convolution of 3D OCT volume and the Gabor filter result in a Gaussian-averaged OCT volume, where one has access to the spatial frequency spectrum along the z-direction at each voxel:


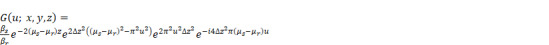

(4)

One can readily see from Eq. (4) that the AC of the sample can be obtained by analysing the phase of 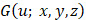
(i.e., 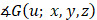), which is a linear function of the spatial frequency 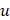. The normalized AC (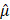) of the sample is then derived by evaluating the slope of 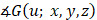
as


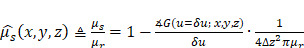

(5)

where 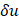
is the frequency sampling interval in the 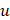
domain. BSC can be obtained by examining the absolute value of 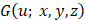
at 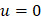
frequency as follows:


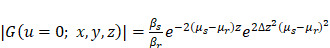

(6)

From Equation (6), the BSC (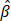) normalized to the reference can be derived as


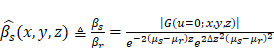

(7)

This method is the first to apply a complex-valued Gabor filter to OCT datasets for simultaneous 3D estimation of AC and BSC. The application of the Gabor filter to OCT datasets results in complex-valued 3D map, and from its magnitude and phase, AC and BSC at specific locations can be measured with high precision. It should be noted that, this dual-parameter analysis has not been explored in tumor spheroid imaging.

We validated the Gabor filter-based AC and BSC extraction method through numerical simulations on a digital phantom and experiments with 0.5%, 1%, 2.5%, and 5% intralipid solutions. The 1/*e*^2^ radii of the Gaussian function were set as ∆x = ∆y = 20.0 μm and ∆z = 6.1 μm, respectively. Numerical analysis demonstrated that our method achieved higher accuracy, with AC and BSC errors of only 0.4% and 4.7%, respectively, than FD method (Figure S4) 42. Our experimental results with intralipid solutions also validated the high accuracy and robustness of our method, yielding an average AC error of 0.8% and high linearity of BSC estimates to intralipid concentrations (R^2^ = 0.99) (**Table S1**, **Figure S1**).

### Statistical Analysis

All data are presented as mean ± standard error. This study was conducted as a pilot study, with each having three replicates. Comparisons between groups were performed using a two-tailed unpaired Student's *t*-test and a one-way ANOVA multiple comparison test. Statistical significance levels were established as *p < 0.05, **p < 0.01, and ***p < 0.001 with the sample counts specified in the figure captions.

## Results and Discussion

### Quantitative Morphological Analysis of BT474 Spheroids via OCT and Bright-field Imaging

**Figure 2:**
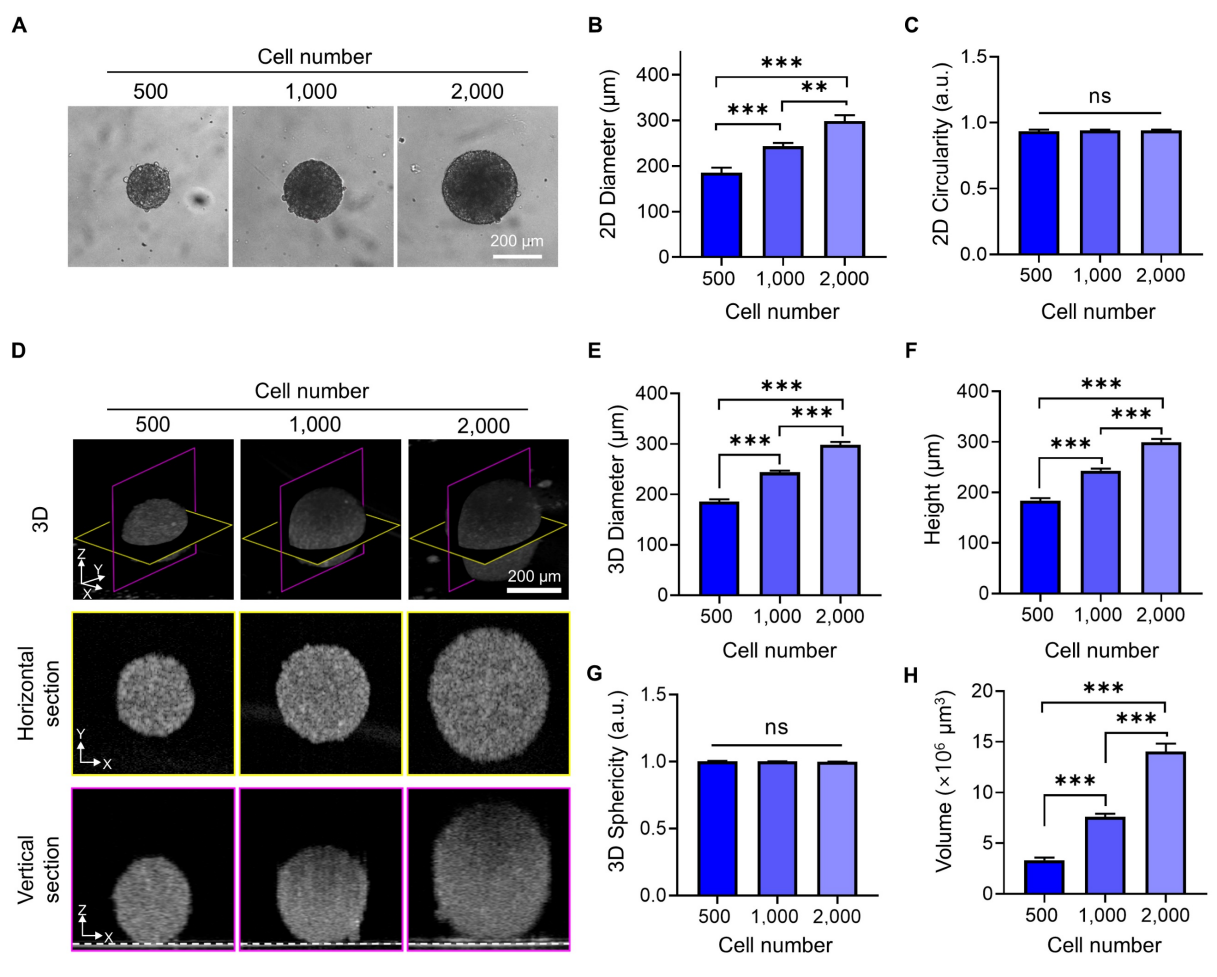
Quantitative morphological analysis of BT474 spheroids using bright-field and OCT imaging. (A) Representative bright-field images of BT474 spheroids formed from 500, 1,000, and 2,000 cells. Scale bar: 200 μm. Quantification of 2D diameter (B) and circularity (C) based on bright-field images. (D) 3D OCT reconstructions of spheroids, with corresponding horizontal (middle row) and vertical (bottom row) cross-sectional views. Section views were acquired at depths of approximately 91 μm, 121 μm, and 149 μm above the plate surface, respectively. The dashed line in the vertical sections indicates the bottom surface of the well plate. Scale bar: 200 μm. (E-F) Quantitative OCT-based measurements of spheroid diameter (E) and height (F), both of which significantly increase with cell number. (G) Sphericity values derived from 3D OCT imaging. (H) Quantification of total spheroid volume, calculated by counting the number of voxels corresponding to the spheroid and converting this to physical units. Statistical analysis was performed using one-way ANOVA with Tukey's post hoc test. ***p < 0.001; ns = not significant; n = 5.

To quantitatively assess how tumor spheroids expand in three dimensions with increasing cell number, we analyzed their diameter, height, volume, and 3D circularity using OCT imaging and compared these metrics with 2D measurements obtained from bright-field imaging. Bright-field images (**Figure 2A, B**) showed that spheroid diameter increased proportionally with the number of seeded cells. The 2D circularity remained consistently close to 1 across all conditions (**Figure 2C**), indicating that the spheroids maintained a symmetrical shape in the horizontal plane. However, this projection-based view could not capture vertical growth or shape distortion along the z-axis.

OCT imaging provided depth-resolved structural information, revealing that spheroids increased not only in diameter but also in height, leading to a substantial increase in overall volume (**Figure 2D-F**). The 3D sphericity remained close to 1 across all conditions (**Figure 2G**), demonstrating that spheroids preserved a highly symmetric and spherical structure even as they grew—a feature not previously quantitatively assessed using OCT 21, 23.

While confocal laser scanning microscopy is widely used for 3D imaging of spheroids and offers high-resolution optical sectioning, it has limitations in capturing full-depth geometry, especially in larger spheroids. This is mainly due to signal attenuation and scattering at shorter wavelengths, which restricts imaging depth and can lead to incomplete reconstruction of the vertical structure of the spheroid (**Figure S5**) 43. These limitations make OCT a valuable tool for capturing the true 3D shape and structural integrity of spheroids without the need for fluorescent staining, providing a clearer and more accurate representation of their morphology.

### Quantitative Correlation of OCT-derived AC and BSC Values with Viability of Drug-treated Tumor Spheroids

Quantitative optical parameters derived from OCT provide valuable insights into tissue microstructure and integrity. These parameters have separately been used to assess apoptotic and pathological changes across various biological tissues 29, 31, 44-50, but the combined metric has never been explored for the analysis of tumor spheroid. To evaluate the validity of our method, we measured the changes in AC and BSC values in tumor spheroids treated with AZD4547, a drug that blocks FGFR signaling 37. In breast cancer cells, this drug is known to trigger apoptosis by damaging mitochondria 37, 51, and the resultant fragments produce heterogeneous environment inside the cells. As a result, we hypothesized that mitochondrial-mediated apoptosis would lead to higher AC and BSC values due to the formation of these small scattering particles.

**Figure 3:**
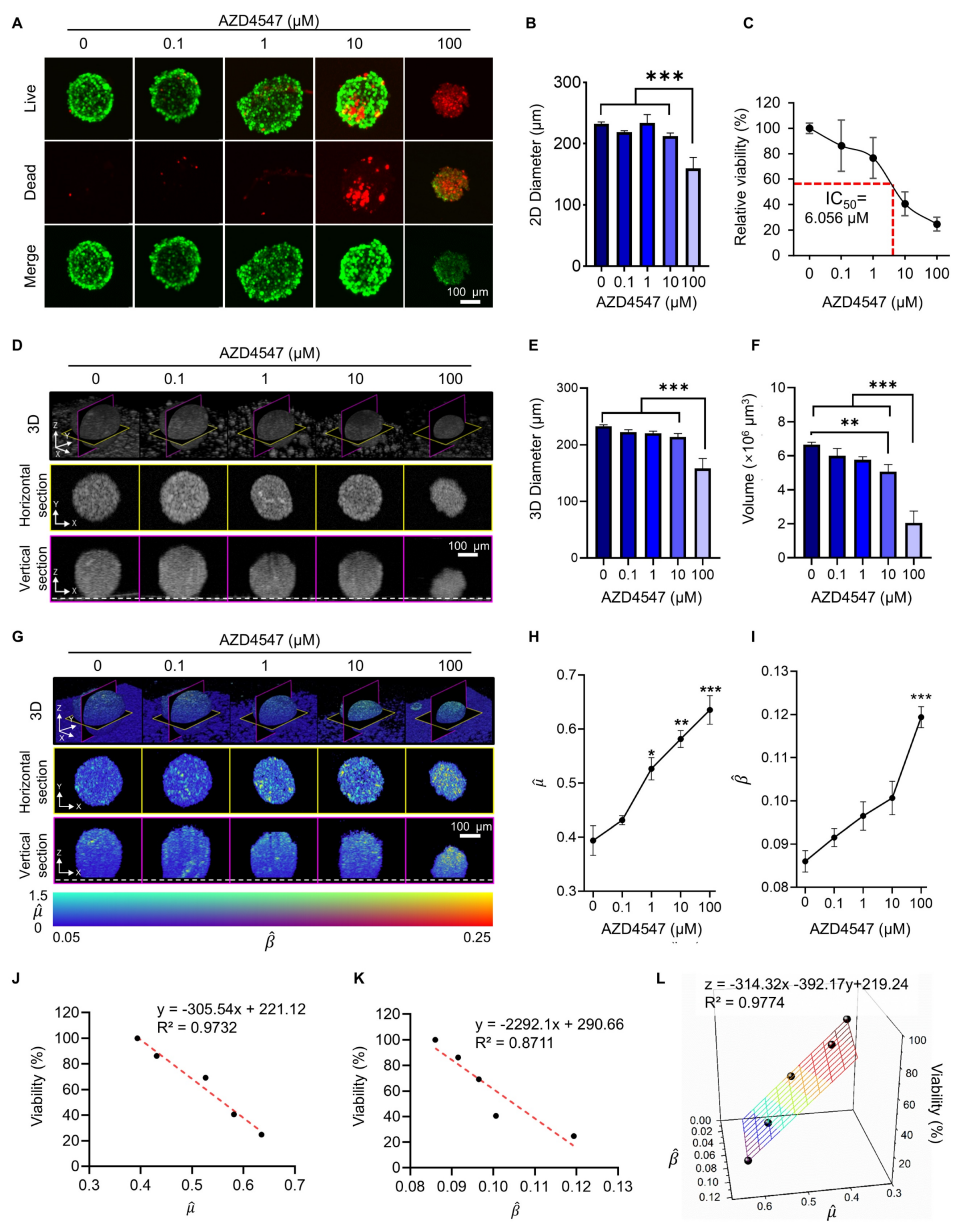
Evaluation of AZD4547-induced apoptosis in BT474 spheroids using OCT-derived AC (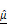) and BSC (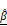) parameters. (A) Representative LIVE/DEAD staining images of BT474 spheroids treated with increasing concentrations of AZD4547 (0-100 μM). Scale bar: 100 μm. (B-C) Quantification of 2D spheroid diameter (B) and relative viability (C), both showing dose-dependent decreases, with an estimated IC₅₀ of 6.1 μM. (D) 3D OCT reconstructions with corresponding horizontal and vertical cross-sections, highlighting morphological changes following AZD4547 treatment. In the vertical sections, the dashed line denotes the bottom surface of the well plate. Scale bar: 100 μm. (E-F) OCT-based quantification of 3D diameter (E) and volume (F), both of which decreased with higher drug concentrations. (G) Volumetric visualization of 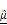
and 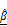, overlaid in 3D and cross-sectional views, showing increased signal intensity with rising AZD4547 dose—indicative of apoptosis-induced structural changes. Scale bar: 100 μm. (H-I) Quantification of 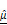
(H) and 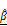
(I) as functions of AZD4547 concentration.(J-K) Linear correlation analyses between spheroid viability and 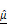
(J) and 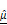
(K), demonstrating strong inverse relationships. (L) 3D multivariate regression model combining 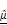
and 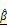, yielding improved predictive correlation with spheroid viability (R² = 0.98). Spheroid volume was calculated by counting voxels corresponding to the spheroid and converting this count to physical units. Statistical analysis was performed using one-way ANOVA with Tukey's post hoc test: **p < 0.01 and ***p < 0.001. n = 3.

BT474 spheroids approximately 200 μm in diameter were treated with increasing concentrations of AZD4547 (0-100 μM). LIVE/DEAD staining showed a dose-dependent reduction in viable cells and an increase in dead cells (**Figure 3A**). At 100 μM, spheroid diameter was significantly reduced, although 2D circularity remained largely unchanged (**Figure 3B**, **Figure S6**), indicating that gross morphology was preserved despite volume loss. Viability analysis revealed a consistent decrease in spheroid viability with increasing drug concentration, with an estimated IC₅₀ of 6.1 μM (**Figure 3C**).

Structural OCT imaging enabled label-free evaluation of 3D spheroid morphology after treatment, revealing dose-dependent reductions in diameter, height, and volume (**Figure 3D-F**). Of note, 3D volume was observed to decrease up to 10 µM of AZD4547, but this decrease was more dramatic at 100 µM. Compared to the untreated control, spheroids treated with 100 µM AZD4547 showed approximately 35% reductions in both height and diameter, and a 69% reduction in volume. These results demonstrate the potential of this approach for *in situ* assessment of drug-induced structural changes.

Corresponding changes were evident in the tomographic AC and BSC images. Features with high AC and BSC (visualized in yellow) become more pronounced with increasing AZD4547 concentrations (**Figure 3G**), indicating enhanced scattering likely due to intracellular degradation and apoptotic processes. Quantitative measurements showed that mean AC values increased with drug dose, reaching a peak of 0.64 for spheroids treated with 100 μM AZD4547 (**Figure 3H**). Similarly, BSC values increased with dose, peaking at 0.12 at the highest concentration (**Figure 3J**). Linear regression analysis revealed strong inverse correlations between relative spheroid viability and both AC and BSC values. The AC parameter showed a particularly strong linear correlation (R² = 0.97; **Figure 3J**), while BSC values correlated with an R² of 0.87 (**Figure 3K**). Combining AC and BSC as dual metrics further improved the correlation (R² = 0.98; **Figure 3L**), indicating superior viability estimation when both parameters were integrated.

The observed optical changes aligned with well-recognized features of apoptosis, such as cellular shrinkage 33, chromatin condensation 52, nuclear fragmentation 34, and mitochondrial swelling 53, along with the release of cytochromes 54. We performed separate fluorescence imaging of BT474 monolayers treated with AZD4547 (**Figure S7**). Upon AZD4547 treatment, Calnexin fluorescence imaging showed condensed perinuclear aggregation, indicating endoplasmic reticulum collapse, while images with MitoTracker revealed dotted, fragmented mitochondrial structures, reflecting loss of membrane potential and mitochondrial disintegration. These organellar derangements are canonical hallmarks of ER-stress-driven, intrinsic apoptosis and precede mitochondrial outer-membrane permeabilization and caspase activation 32, 34-36. Consequently, the reticular architecture of organelles is disrupted, increasing cytoplasmic heterogeneity and RI discontinuities, thereby enhancing the BSC in treated spheroids. The sensitivity of these OCT-derived parameters to apoptotic microstructural alterations highlighted their potential as label-free indicators of cell death 55, 56.

### CAR T-induced Dynamic Structural and Viability Changes in Tumor Spheroids

**Figure 4:**
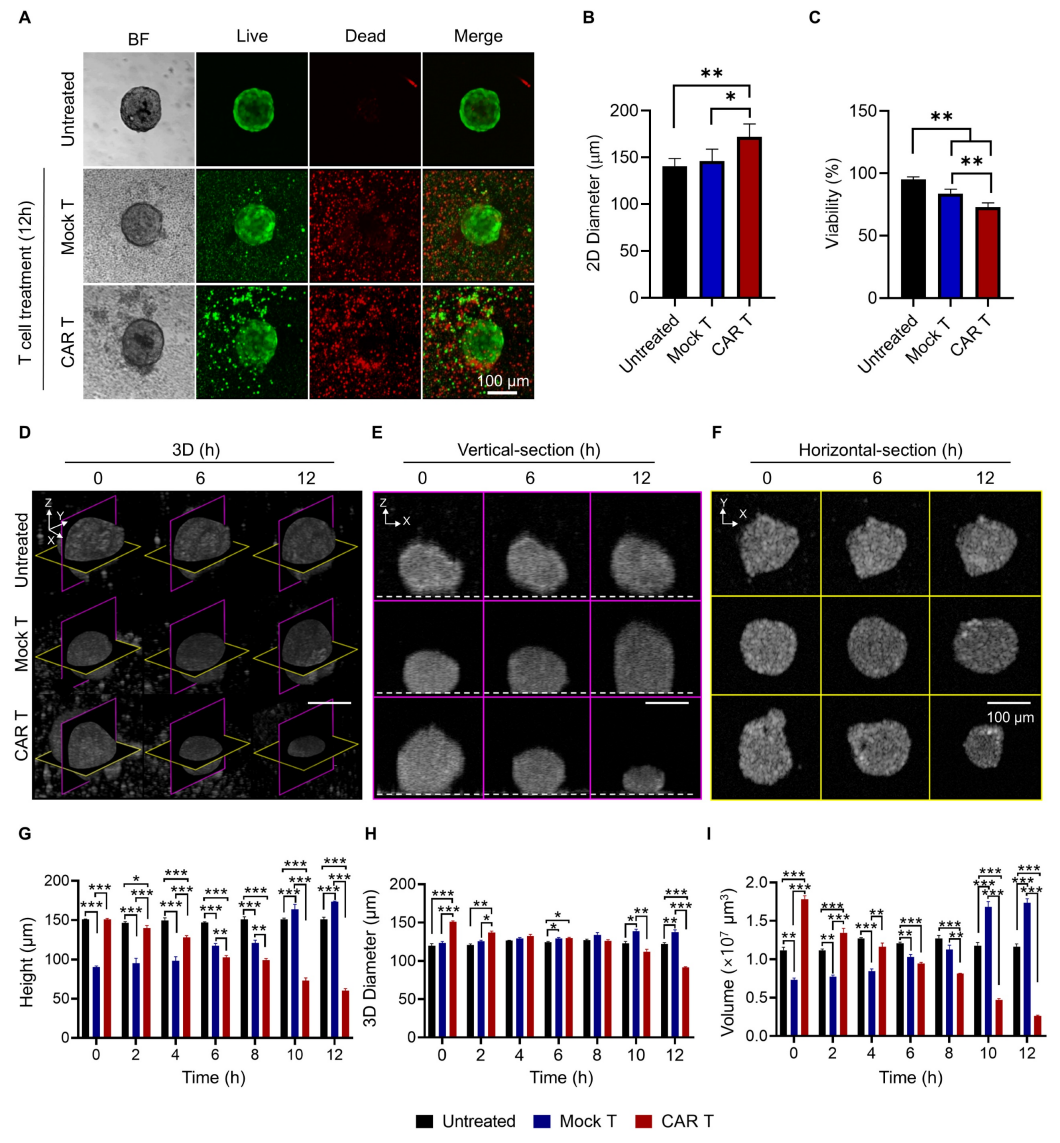
Longitudinal OCT imaging reveals dynamic morphological changes in tumor spheroids following CAR T cell treatment. (A) Representative bright field and fluorescence LIVE/DEAD images of BT474 spheroids 12 h after treatment with mock or HER2 CAR T cells. Scale bar: 100 μm. (B-C) Quantification of 2D spheroid diameter (B) and viability (C). CAR T cell treatment led to significant viability loss despite a slight increase in diameter, likely due to T cell accumulation on the spheroid surface. One-way ANOVA with Tukey's post hoc test: *p < 0.05; **p < 0.01; and ***p < 0.001. n = 5. (D-F) OCT-based time-lapse imaging of spheroids at 0, 6, and 12 h post-treatment, showing 3D reconstructions (D), vertical (E), and horizontal (F) sections. Dashed lines in the vertical sections indicate the location of the well plate bottom. Scale bar: 100 μm. (G-I) Quantification of spheroid height (G), 3D diameter (H), and volume (I) over time. CAR T cell treatment caused significant reductions in all structural parameters, while mock T cell treatment induced minor structural changes. One-way ANOVA with Tukey's post hoc test: *p < 0.05; **p < 0.01; and ***p < 0.001. n = 4.

To evaluate the cytotoxic effects of HER2 CAR T cells, LIVE/DEAD staining was performed on tumor spheroids. LIVE/DEAD imaging revealed extensive cell death in the CAR T cell-treated group compared to the mock T cell group, confirming the strong cytotoxic activity of HER2 CAR T cells (**Figure 4A**). However, due to the limited imaging depth of confocal microscopy (~80 µm), a comprehensive assessment of structural changes within the spheroids was not feasible (**Figure S8**) 57.

Interestingly, the overall diameter of CAR T cell-treated spheroids increased despite the observed cell death. This was likely due to peripheral binding and accumulation of CAR T cells, which disrupted and loosened the spheroid structure (**Figure 4B**) 38, 58. This finding suggests that diameter-based measurements alone may misrepresent actual structural alterations.

Viability analysis using LIVE/DEAD staining showed a significant reduction in the CAR T cell-treated group compared to the mock group (**Figure 4D**). However, this result may be confounded by the presence of dead CAR T cells, potentially leading to an underestimation of true spheroid viability 59. Although washing steps can theoretically remove loosely bound and dead CAR T cells from the spheroid surface, they carry a high risk of spheroid loss, especially when using non-adhesive microplate systems 38. Additionally, LIVE/DEAD staining is an endpoint assay and does not allow for longitudinal monitoring of morphological changes. These limitations highlight the need for imaging methods capable of providing non-invasive, 3D, time-resolved assessments of CAR T cell-tumor interactions.

To address these limitations, OCT was employed to monitor structural changes in a time-resolved and volumetric manner. Vertical-section, horizontal-section, and 3D OCT images revealed a marked reduction in spheroid size following HER2 CAR T cell treatment (**Figure 4E-G**). Quantitative analysis confirmed that spheroid height, diameter, and volume significantly decreased in the CAR T cell-treated group compared to both untreated and mock-treated groups (**Figure 4H-J**). This volumetric reduction underscores the potent cytotoxic effect of HER2 CAR T cells, whereas the mock T cell-treated spheroids exhibited an increase in height over time, likely due to surface accumulation of T cells.

These results demonstrate that OCT enables comprehensive, longitudinal assessment of CAR T cell-induced cytotoxicity in tumor spheroids, capturing dynamic, volumetric changes that are challenging to evaluate using conventional imaging techniques.

### OCT-based Measurement of Apoptosis through BSC and AC Correlation with PI Expression in Tumor Spheroids

**Figure 5:**
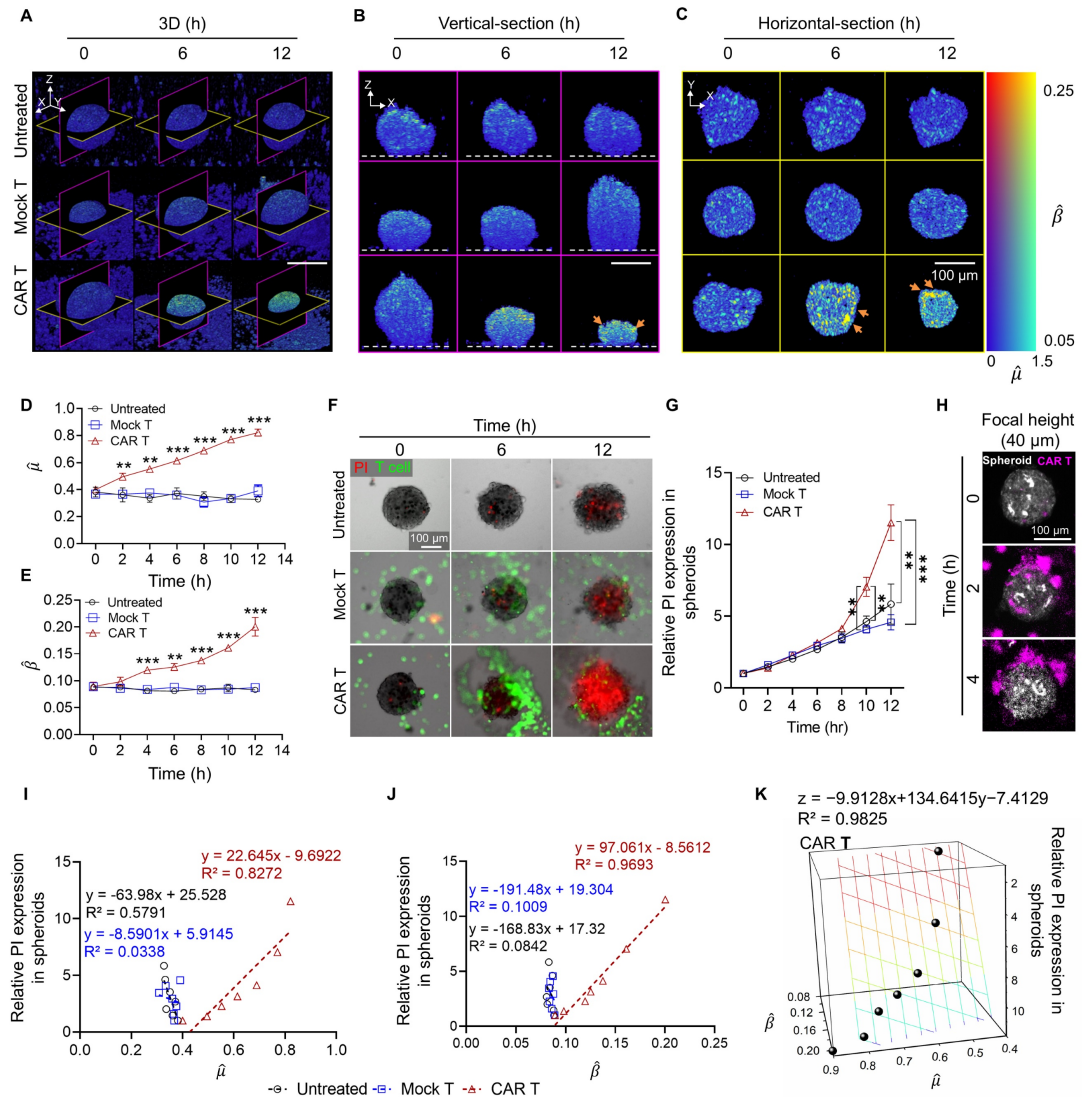
OCT-derived AC (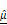) and BSC (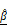) parameters correlate with PI-based apoptosis in CAR T cell-treated tumor spheroids. (A-C) 3D OCT reconstructions (A), vertical (B), and horizontal (C) sections of BT474 spheroids treated with mock or HER2 CAR T cells for up to 12 h. Pseudo color maps represent spatial distribution of 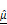
and 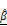. In vertical views, the dashed line indicates the position of the well plate bottom. Scale bar: 100 μm. (D-E) Time-dependent quantification of 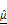
(D) and 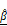
(E), showing significant increases in the CAR T group, indicating progressive structural degradation and increased scattering. One-way ANOVA with Tukey's post hoc test: **p < 0.01; ***p < 0.001. n = 3. (F) Representative fluorescence images showing PI-based cell death (red) and T cells (green) over 12 h. Scale bar: 100 μm. (G) Quantification of relative PI expression in spheroids. PI signal increased significantly after 10 h in the CAR T group. One-way ANOVA with Tukey's post hoc test: **p < 0.01; ***p < 0.001; and ns= not significant. n = 9. (H) Confocal z-stack projections showing CAR T cell (magenta) infiltration into the outer 10-20 μm of spheroids (white). Scale bar: 100 μm (I-J) Linear correlation analysis between PI intensity and OCT-derived 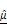
(I) and 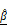
(J). Strong inverse correlations were observed in the CAR T group. (K) Multivariate regression analysis using both 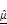
and 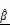
to predict PI signal, showing improved correlation (R² = 0.9825) in CAR T-treated spheroids.

We investigated whether OCT-derived AC and BSC values could accurately quantify the cytotoxic effects of HER2 CAR T cells in tumor spheroids. 3D reconstructions of tumor spheroids (**Figure 5A**) showed significantly elevated AC and BSC values in HER2 CAR T cell-treated spheroids compared to untreated and mock T cell-treated groups, with magenta and yellow slices indicating vertical and horizontal cross-sections, respectively. Vertical and horizontal section views acquired ~64 µm above the plate surface (**Figure 5B, C**), further confirmed the increased AC and BSC values in CAR T-treated spheroids relative to controls.

By 12 h post-treatment, CAR T cell-treated spheroids reached a normalized AC value of approximately 0.82, more than twice that of mock-treated (0.39) and untreated (0.33) spheroids (**Figure 5D**). Similarly, BSC values exhibited an exponential increase over time, reaching 0.20 at 12 h, while the mock and untreated groups-maintained BSC values below 0.10 (**Figure 5E**). These findings may be explained by structural changes and cellular fragmentation following T cell introduction 60. During the apoptosis, chromatin condensation, cytoplasmic compaction, and organelle fragmentation have been reported to increase RI heterogeneity and scattering signals 32, 34-36. Furthermore, the high AC and BSC values in CAR T-treated spheroids, relative to AZD4547, are consistent with rapid, extensive ultrastructural disruption from perforin/granzyme-mediated cytotoxicity, thereby increasing optical heterogeneity over short timescales 40, 61. Given that light attenuation in biological tissues at near-infrared wavelengths is dominated by scattering rather than absorption, increased attenuation primarily reflects increased RI heterogeneity and scatterer density. Additionally, according to scattering theory, for a particle with its size (r) larger than the incident wavelength, BSC is inversely proportional to particle size (β ∝ 1/r). The observed increase in BSC values for CAR T-treated spheroids may therefore be attributed to cellular fragmentation, leading to the formation of smaller scatterers within the imaging volume.

To assess whether the cytotoxic effects observed through AC and BSC measurements were associated with apoptosis, PI staining was used to selectively identify apoptosis of cancer cells (**Figure 5F**) 62. The PI fluorescence intensity began to rise at 6 h post-treatment and showed a pronounced increase by 10 h, closely aligning with the temporal trends observed in AC and BSC values (**Figure 5G**). Furthermore, AC and BSC were sensitive to early indicators of immune cell engagement or stress responses (**Figure S9**). AC and BSC values significantly increased during the early hours (2-6 h), even in the absence of detectable PI expression. Confocal z-projection imaging revealed that HER2 CAR T cells (magenta) infiltrated approximately 10-20 µm into the spheroid periphery (white) (**Figure 5H**). Notably, OCT-derived AC and BSC also exhibited significant elevation in the same peripheral zone (**Figure 5A-C**), suggesting that CAR T cell-mediated cytotoxic activity may lead to localized structural and optical changes in the spheroid periphery. The peripheral-to-central propagation of optical property changes likely reflects immune infiltration gradients, serial killing behavior, and spatial heterogeneity within spheroids. CAR T cells initially accumulated at the periphery and gradually infiltrated inward 63, a pattern mirrored by the OCT-based dual-parameter analysis (**Figure 5C**). The sequential cytotoxic activity of CAR T cells induced a wave of apoptosis from the periphery to the center, consistent with increasing PI signals despite stable T cell numbers at the core of spheroids from the beginning (**Figure S10, Movie 1-3**). As shown in the CAR T treated spheroid in Movie 3, green fluorescence labelled CAR T were observed similarly from 6 to 12 hr. The 3D AC and BSC tomograms in Movie 4 further confirm that features of high AC and BSC originate at the periphery and progress toward the core only in CAR T-treated spheroids (**Movie 4**).

Consistent with the results observed for AZD4547 treatment, linear regression analysis demonstrated strong inverse correlations between spheroid viability and both AC (R² = 0.83; **Figure 5I**) and BSC (R² = 0.97; **Figure 5J**). When AC and BSC were combined as a dual-parameter predictor, the correlation improved further (R² = 0.98; **Figure 5K**), reinforcing their combined predictive value for assessing cell viability.

Previous studies have shown that CAR T cells can infiltrate tumor spheroids and induce extensive apoptosis—effects not fully captured by gross changes in tumor size alone 48. AC and BSC measurements provided sensitive means to detect immune cell-induced apoptosis, as demonstrated by increased signal intensity in regions where CAR T cells were observed. This spatial correlation suggests localized cytotoxic interactions between CAR T cells and cancer cells. In this context, tomographic AC and BSC imaging proves particularly valuable, enabling the quantitative assessment of AC and BSC values across the entire spheroid and offering a comprehensive evaluation of cell death and CAR T cell distribution.

## Conclusions

This study demonstrated the utility of tomographic AC and BSC imaging as an effective, noninvasive tool for evaluating CAR T cell cytotoxicity in tumor spheroids. By providing 3D morphological and optical information on spheroid structures, this method enabled precise and quantitative assessment of cytotoxic effects without the need for perturbative procedures such as staining. Volumetric AC and BSC features showed strong correlations with spheroid viability, with a combined coefficient of determination (CoD) of 0.98. This dual-parameter approach outperformed individual AC-only (CoD ~ 0.83) and BSC-only (CoD ~ 0.97) analyses when compared against fluorescence-based viability assays. Our results indicate that CAR T cells induced substantial structural disruption and cell death in spheroids, as evidenced by increased AC and BSC values and decreased viability. In contrast, mock T cells did not cause significant changes in spheroid morphology or viability, highlighting the specificity and potency of CAR T cell activity.

Our imaging results revealed elevated AC values throughout the entire volume of CAR T cell-treated spheroids, accompanied by granular regions of high BSC values predominantly in the periphery. These observations were consistent with fluorescence-based analyses, which indicate that CAR T cell infiltration initiates at the spheroid surface. Most biological cells and tissues exhibit minimal absorption in the near-infrared spectral range. Thus, the observed changes in AC and BSC can be reliably interpreted using light scattering theory. According to Mie scattering theory, elevated AC values could result from increased scatterer density, enhanced RI mismatches, and greater structural irregularities within the spheroid. BSC values tend to rise when structures become more layered or when the size and shape of the scatterers change. For instance, for particles larger than the incident wavelength, a reduction in scatterer size leads to an increase in BSC.

These physical insights help explain the elevated AC and BSC values observed following CAR T cell treatment. Specifically, apoptosis induced by CAR T cells triggers distinctive morphological changes, such as mitochondrial swelling, cytoplasmic reorganization 32, nuclear condensation (pyknosis) and cellular fragmentation (karyorrhexis), which generate a denser and more heterogeneous microenvironment 33, 34. Numerous *in vitro* microscopic studies have shown that apoptotic cells display highly perturbed membranes and small granular structures or blebs with high RI values 35, 36. These fragmented intracellular components and debris form a more highly scattering volume, likely accounting for the observed increases in AC and BSC. These changes were initially detected at the spheroid periphery and progressively spread inward, indicating CAR T cell infiltration and apoptosis progression. This pattern was also corroborated by fluorescence imaging, which revealed spatially progressive cell death from the spheroid surface inward. The observed high sensitivity of combined AC and BSC to these early intracellular events is particularly advantageous for detecting the onset of immune-mediated cytotoxicity, as evidenced by high correlation with cell viability results. We also conducted separate AC and BSC imaging experiments on tumor spheroids treated with nigericin, a necrosis-inducing agent. Our results found marked decreases in AC and BSC values of tumor spheroids associated with necrotic features, which represents distinct and opposing responses compared to apoptosis.

In our experiments, we intentionally underfilled the microscope objective to achieve a large DoF for imaging the entire spheroid with a uniform transversal resolution. Even with this implementation, normalization with the measurement of a reference (i.e., an intralipid solution in our case) was still required to eliminate the influence of other system parameters on our analysis. However, this implementation compromises the lateral resolution. Various methods to increase the lateral resolution have been developed, including deconvolution 64, 65, digital focusing 66, 67, and adaptive optics 68, 69. The implementation of such methods would enable OCT imaging and cytotoxicity evaluation with a higher spatial resolution. For instance, OCT imaging with a needle-like beam is a representative hardware-based solution that enables structural imaging with isotropic resolution 70-73. One of our future endeavors is to implement such a strategy for the high-resolution imaging and more accurate estimation of AC and BSC values in 3D space.

Moreover, as in other OCT-based studies, speckle noise is a critical challenge in the accurate determination of AC and BSC values. Our 3D Gabor-based approach applied Gaussian filtering to reduce speckle noise significantly. Nevertheless, other methods—ranging from optical hardware improvements to advanced post-processing algorithms—may further mitigate speckle noise 74-77. Incorporating such techniques would enable more detailed visualization of cellular features in tumor spheroids and improve the precision of AC and BSC quantification.

Deep learning-based approaches may also be explored to identify structural features associated with apoptosis in OCT images. Prior studies have achieved expert-level segmentation of retinal OCT data using models such as fully convolutional networks combined with Gaussian process regression 78, 79. Applying similar machine learning models to the automated, rapid, and reliable detection of apoptotic signatures represents a promising direction for future research. Although both apoptosis and necrosis contribute to cell death, their underlying mechanisms differ, which may result in distinct AC and BSC profiles. During apoptosis, mitochondrial swelling and increased reactive oxygen species levels cause structural changes that increase AC 32. In contrast, necrosis typically results in a loss of scattering structures due to cell rupture and disintegration, leading to a decrease in AC 80. Future studies will focus on applying deep learning to enhance the segmentation of cell death regions and differentiate between apoptotic and necrotic processes.

Future research should investigate more complex spheroid models that include stromal components, such as cancer-associated fibroblasts, to better replicate the tumor microenvironment and evaluate immunomodulatory effects on CAR T cell efficacy, building on this foundation. Incorporating these elements will yield more physiologically relevant models. Furthermore, applying tomographic AC and BSC imaging to *ex vivo* tissue samples, in conjunction with CAR T immunoassays, will help evaluate the clinical translatability of this approach and its potential to inform personalized treatment strategies.

## Supplementary Material

Supplementary figures and table.

Supplementary movie 1.

Supplementary movie 2.

Supplementary movie 3.

Supplementary movie 4.

## Abbreviations

3D: three-dimensional
2D: two-dimensional
AC: attenuation coefficient
ANOVA: analysis of variance
AO: adaptive optics
BSC: backscattering coefficient
CAR: chimeric antigen receptor
CD3/CD28: cluster of differentiation 3 and 28
CV: coefficient of variation
CoD: coefficient of determination
DoF: depth of focus
EthD-1: ethidium homodimer-1
ER: endoplasmic reticulum
FBS: fetal bovine serum
FGFR: fibroblast growth factor receptor
HER2: human epidermal growth factor receptor 2
IC₅₀: half maximal inhibitory concentration
IL-2: interleukin-2
LIV: logarithmic intensity variance
MC: methyl cellulose
MEM: Minimum Essential Medium
OCT: optical coherence tomography
PDMS: polydimethylsiloxane
PFA: paraformaldehyde
PI: propidium iodide
PSF: point spread function
RI: refractive index
R²: coefficient of determination
RPMI: Roswell Park Memorial Institute
RT: room temperature
SLD: superluminescent diode
STR: short tandem repeat
TME: tumor microenvironment
WST: water-soluble tetrazolium salt
normalized attenuation coefficient
normalized backscattering coefficient
