# Supplementary material for: Dual-parameter tomographic imaging of attenuation and backscattering coefficients for quantitative evaluation of immune cell-mediated cytotoxicity in tumor spheroids
Source: Theranostics. 2025 Aug 22;15(18):9399–414. doi: 10.7150/thno.118722 (PMC12486019; doi:10.7150/thno.118722)
Supplement: Supplementary file 1 — Supplementary figures and table. [file thnov15p9399s1.pdf]

Supporting Information

Dual-Parameter Tomographic Imaging of Attenuation and Backscattering for Quantitative Evaluation of Immune Cell-Mediated Cytotoxicity in Tumor Spheroids

Seokgyu Han[1,†], Ingyoung Kim[2,†], Baekcheon Seong[2], Woovin Kim[2], Hongseong Kim[2], Sein Kim[3], Chulmin Joo[2], and Sungsu Park[1,4,5*]*

[1] School of Mechanical Engineering, Sungkyunkwan University, Suwon, 16419, Republic of Korea

[2] Department of Mechanical Engineering, Yonsei University, Seoul, 03722, Republic of Korea

[3] Department of Biomedical Engineering, Sungkyunkwan University, Suwon 16419, Republic of Korea

[4] Institute of Quantum Biophysics (IQB), Sungkyunkwan University, Suwon, 16419, Republic of Korea

[5] Department of Metabiohealth, Sungkyunkwan University, Suwon, 16419, Republic of Korea

E-mail: cjoo@yonsei.ac.kr

E-mail: nanopark@skku.edu

[†] S. Han, I. Kim contributed equally as first authors.

A. Experimental comparison of AC and BSC measurement performance: Gabor-based vs. log-and-fitting and FD methods

Table S1 | Quantitative comparison of normalized AC ($\hat{\mu}$) and BSC ($\hat{\beta}$) measurements on intralipid solution of various concentrations (i.e., 0.5%, 1%, 2.5%, 5%). Gabor-based, log-and-fitting (LF), and FD-domain methods were used to obtain normalized AC and BSC values. Note that actual AC values of the solutions were separately measured with a spectrophotometer (V-650, JASCO, USA), and normalized by that of 1% intralipid solution.

Method		Intralipid 0.5%	Intralipid 1%	Intralipid 2.5%	Intralipid 5%
**Spectrophotometry (V-650)**	$\hat{\mu}$	0.530	1.000	2.592	5.541
	$\hat{\beta}$	N/A	N/A	N/A	N/A
**Gabor-based**	$\hat{\mu}$	**0.533±0.004**	**1.002±0.006**	**2.666±0.002**	**5.554±0.004**
	$\hat{\beta}$	**0.544±0.010**	**0.999±0.007**	**1.696±0.004**	**3.310±0.009**
**Log-and-fitting[1] (LF)**	$\hat{\mu}$	0.501±0.083	1.385±0.111	2.304±0.390	4.875±0.357
	$\hat{\beta}$	N/A	N/A	N/A	N/A
**Fourier domain[2] (FD)**	$\hat{\mu}$	0.559±0.026	1.016±0.019	2.794±0.011	5.561±0.038
	$\hat{\beta}$	N/A	N/A	N/A	N/A

[1] Kut, Carmen, et al., "Detection of human brain cancer infiltration ex vivo and in vivo using quantitative optical coherence tomography." Science Translational Medicine 7.292 (2015): 292ra100-292ra100.

[2] Yuan, Wu, et al., "Robust and fast characterization of OCT-based optical attenuation using a novel frequency-domain algorithm for brain cancer detection." Scientific Reports 7.1 (2017): 44909.

We conducted quantitative comparisons of AC and BSC detection performance of our Gabor-based method against previously reported schemes [1, 2]. OCT B-scan imaging (composed of 500 A-lines) was performed on intralipid solutions of various concentrations (0.5%, 1%, 2.5%, and 5%), and normalized AC and BSC for the solutions were estimated via the Gabor-based, log-and-fitting, and FD-domain methods (Table S1). The actual AC values for the solutions were separately measured using a spectrophotometer (V-650, JASCO, USA), but BSC measurements were not available in spectrophotometer, and LF and FD methods.

Our Gabor-based method demonstrated superior accuracy in the AC estimation over all the samples with an average relative error of 0.8%. In contrast, LF and FD approaches yielded average errors of 18.1% and 3.1%, respectively. The larger error in the LF measurements is attributable to its sensitivity to few-scattering effects and to surface-detection inaccuracies, which are exacerbated as the fitted depth range increases [2].

Since the actual BSC values are not available in spectrometry, we used Eq. 7 to estimate BSC with the measured AC values, and evaluated its linearity and precision as a function of intralipid concentration. As can be seen clearly in Figure S1, our Gabor-based method features a high linearity with $R^2 = 0.99$. These findings validate the proposed Gabor-based approach as a highly accurate and noise-robust method for the simultaneous extraction of AC and BSC in OCT imaging.

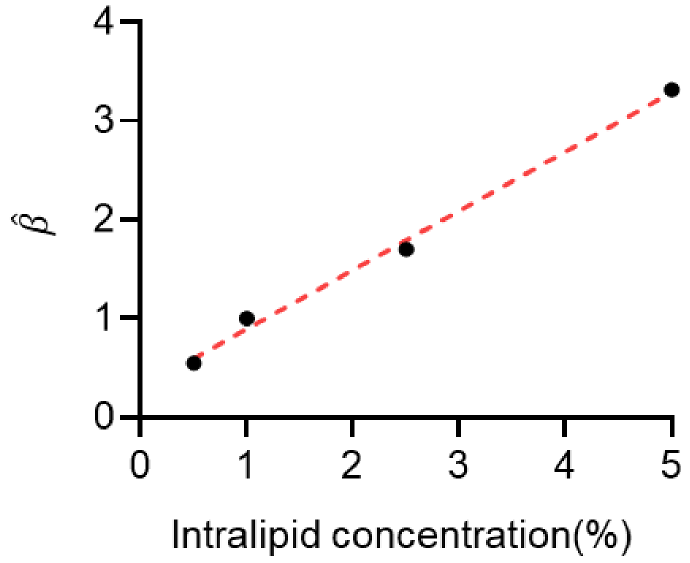

Figure S1. Normalized back-scattering coefficient ($\hat{\beta}$) as a function of intralipid solutions. The Gabor-based method demonstrates a great linear relationship with intralipid concentration, with a high coefficient of determination (R^2 = 0.99).

B. OCT setup and image pre-processing

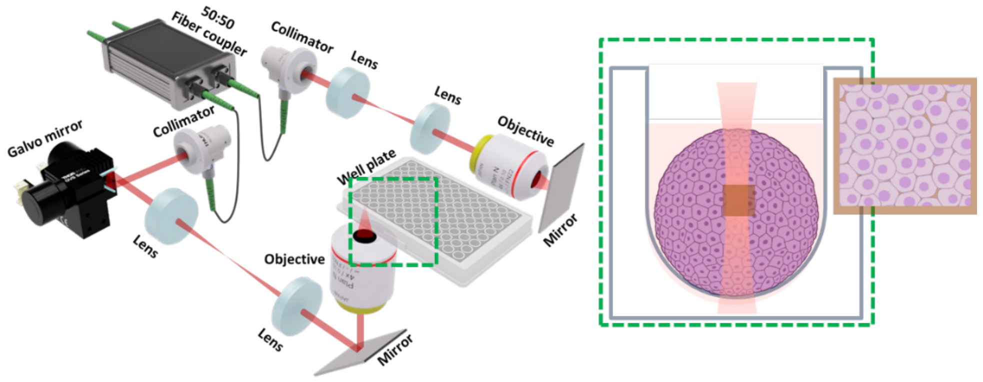

Figure S2. Schematics of OCT.

Our OCT instrument employed a broadband light source (M-T-850-HP-I, SUPERLUM, Carrigtwohill, Ireland) with a central wavelength of 850 nm and a spectral bandwidth of 100 nm (Figure S2). Light from the source was coupled into a 50:50 fiber-based beam splitter (TW850R5A2, Thorlabs), which divided the beam into reference and sample arms. In the sample arm, the beam was focused onto the specimen using a microscope objective (PLN 4X/0.1, Olympus, USA). To enable structural imaging of tumor spheroids with a long depth of focus, we underfilled the probe beam at the back aperture of the objective. Backscattered light from the specimen was recoupled into the fiber and combined with light from the reference arm to produce interference spectra, which were captured by a spectrometer (Cobra-S 800, Wasatch Photonics, USA) at an A-line rate of 50 kHz.

Following acquisition, the DC component—estimated by averaging the spectra over A-lines in the B-scan—was subtracted to generate zero-mean interferograms. The spectra were then zero-padded by a factor of four [1]. To correct for dispersion mismatch between the sample and reference arms, we placed a mirror in the sample arm and measured the nonlinear phase term (i.e., dispersion) of the corresponding interference spectrum. The spectra obtained from the specimen were subsequently multiplied by the complex conjugate of the estimated dispersion to compensate for phase distortions. After these preprocessing steps, the spectra was Fourier transformed to obtain A-line information [2, 3].

We then used measurements obtained with a sample mirror at various depths to correct for OCT signal decay due to finite spectral sampling. In addition, the A-line data were normalized by that of a reference sample (i.e., 1% intralipid solution) to compensate for system-related errors such as PSF distribution, thereby improving the accuracy of attenuation coefficient (AC) and backscattering coefficient (BSC) measurements [1, 3].

C. AC measurement of reference solution using spectrophotometer

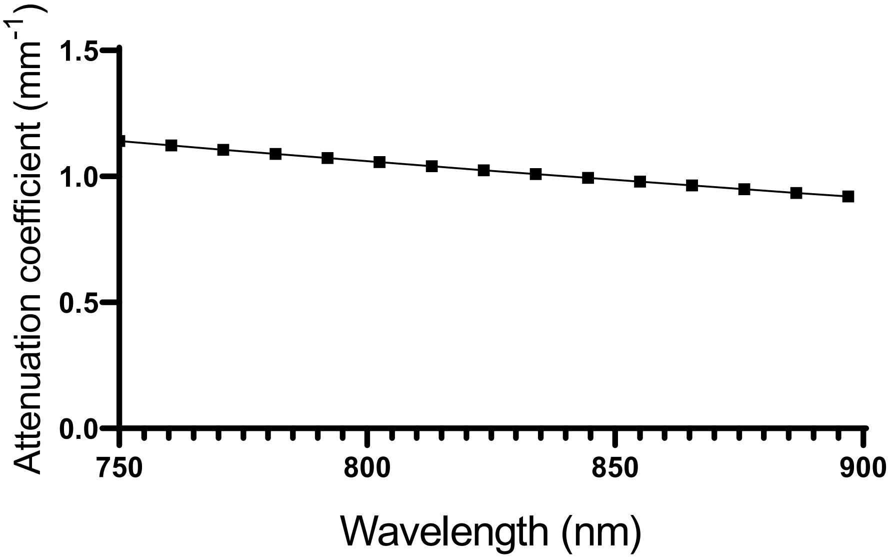

Figure S3. The attenuation coefficient (AC) of a 1% intralipid solution was measured using a UV/VIS spectrophotometer (V-650, JASCO, USA).

The attenuation coefficient (AC) of a 1% intralipid solution was experimentally determined and used as a reference for our Gabor-based AC and BSC estimation. To prepare the sample, a 20% intralipid solution (MFCD00287215, Sigma-Aldrich, USA) was diluted with pure water (MFCD00011332, Sigma-Aldrich, USA) at a ratio of 0.5:9.5. The sample was placed in a quartz cuvette with a thickness of 10 mm (S18-77-536, Starna Cells Inc., USA), and the transmission spectrum of the solution was measured using a UV/VIS spectrophotometer (V-650, JASCO, USA).

The AC was calculated using the Beer–Lambert law, as described by the following equations:

$$T = \frac{I(d)}{I_0} = e^{-\mu d} \tag{1}$$

where I_0 is the incident light intensity, $I(d)$ is the transmitted intensity through the solution with a thickness of d, T is the measured transmittance, and μ is AC. The AC of the 1% intralipid solution at 850 nm—the central wavelength of our OCT system—was determined to be approximately 1 mm^{-1} (Figure S3).

D. Numerical validation of Gabor-based AC and BSC extraction

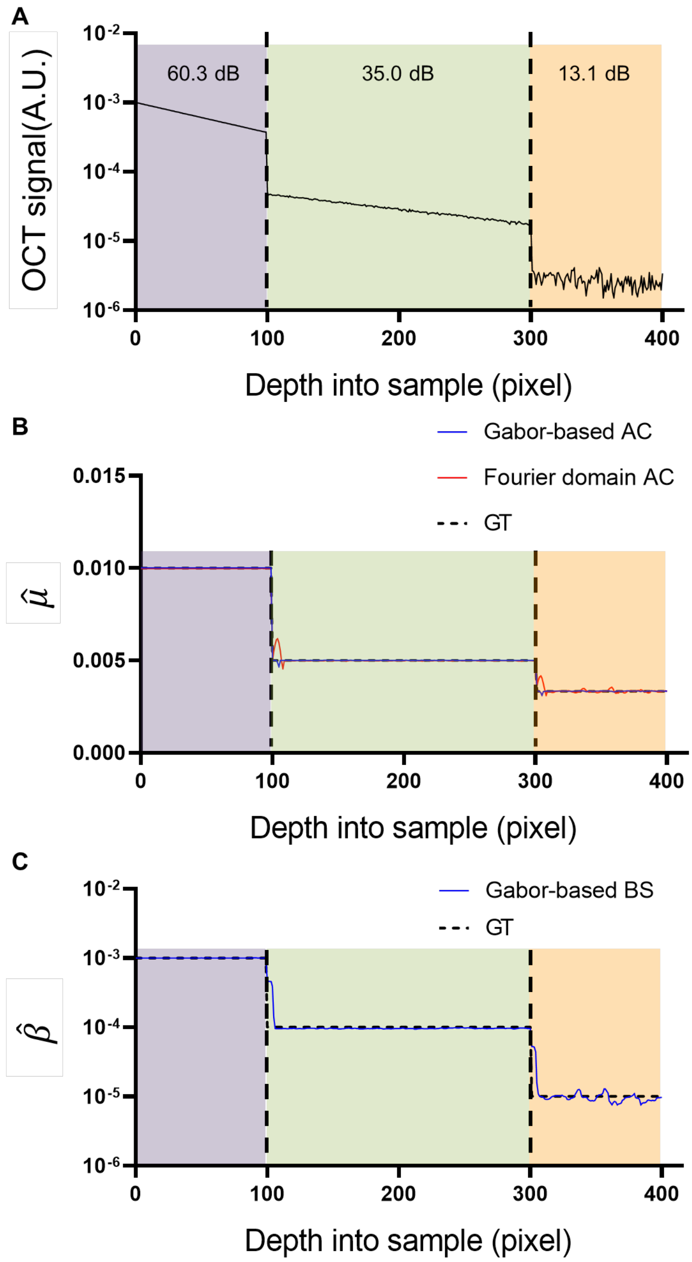

Figure S4. (A) Simulated OCT A-line information for a scattering phantom with depth-dependent AC and BSC distributions. Three depth regions with varying SNR levels—60.3 dB (purple), 35.0 dB (green), and 13.1 dB (orange)—are considered. (B) ACs estimated from the Gabor- (blue line) and the Fourier-domain (FD) methods (red line), compared with the ground-truth information (dashed black line). (C) Comparison of BSCs measured with

the Gabor-based method (blue line) and the ground truth (dashed black line), segmented by the corresponding depth regions with various SNRs in (A).

We performed a numerical validation of our Gabor-based estimation for AC and BSC extraction using a scattering phantom with depth-dependent AC and BSC distributions (Figure S4A). The Gabor filter was applied to the OCT A-line information (Figure S4A) to estimate both AC and BSC. To evaluate performance under various noise levels, we divided the phantom into three segments with distinct signal-to-noise ratios (SNRs): 60.3 dB (purple), 35.0 dB (green), and 13.1 dB (orange).

For comparison, AC was also estimated using the Fourier-domain (FD) method—a fast and robust method for optical attenuation mapping in OCT [2]. Since the FD method does not provide BSC values, it was used solely for comparison of AC estimates. As shown in Figure S4B, both methods accurately estimated AC at high SNR (60.3 dB), with relative errors of 0.2%. However, at lower SNRs, the Gabor-based scheme demonstrated greater robustness: in the 35.0 dB region, relative errors in the AC determination were 0.2% (Gabor) and 0.3% (FD), while in the 13.1 dB region, errors increased to 0.4% (Gabor) and 1.7% (FD). These results suggest the improved robustness of the Gabor-based method in noisy conditions [4]. Figure S4C compares BSC values obtained from the Gabor-based method against ground truth. The relative BSC errors were 0.1%, 4.0%, and 9.9% for the 60.3 dB, 35.0 dB, and 13.1 dB regions, respectively.

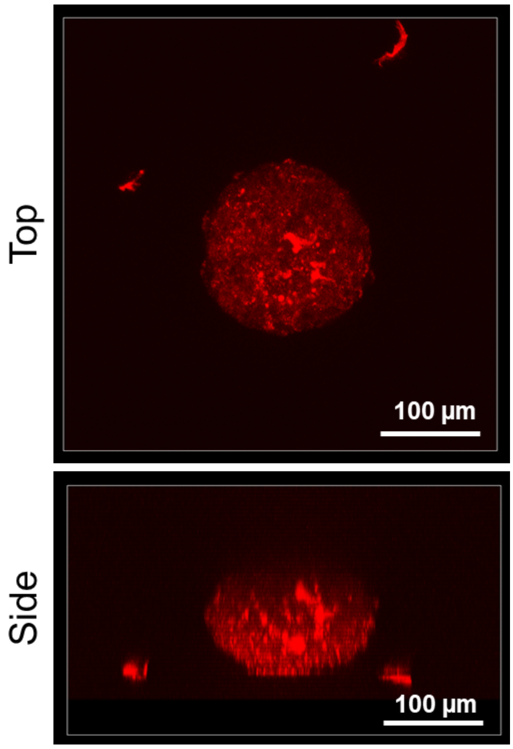

Figure S5. Top and side view of red fluorescence-labeled BT474 spheroids obtained by confocal microscopy (Scale bar = 100 µm).

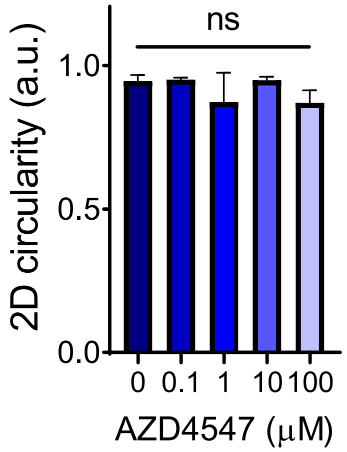

Figure S6. Two-dimensional (2D) circularity of BT474 spheroids treated with different concentrations of AZD4547. Two-tailed, unpaired Student's *t*-test: ns, not significant. Spheroid number n = 3.

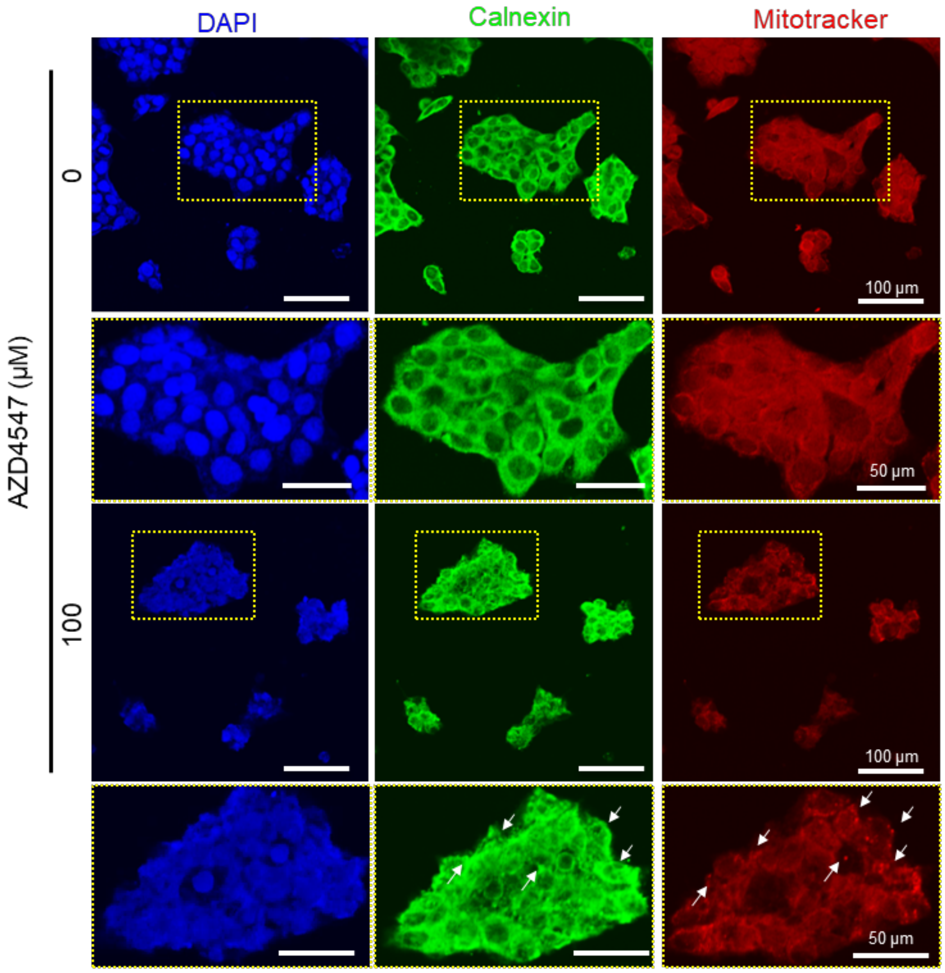

Figure S7. Immunofluorescent images of BT474 cells treated with 100 μM of AZD4547 showing ER (calnexin, green) and mitochondrial (MitoTracker, red) fragmentation. Scale bar = 100 μm and 50 μm for magnified images.

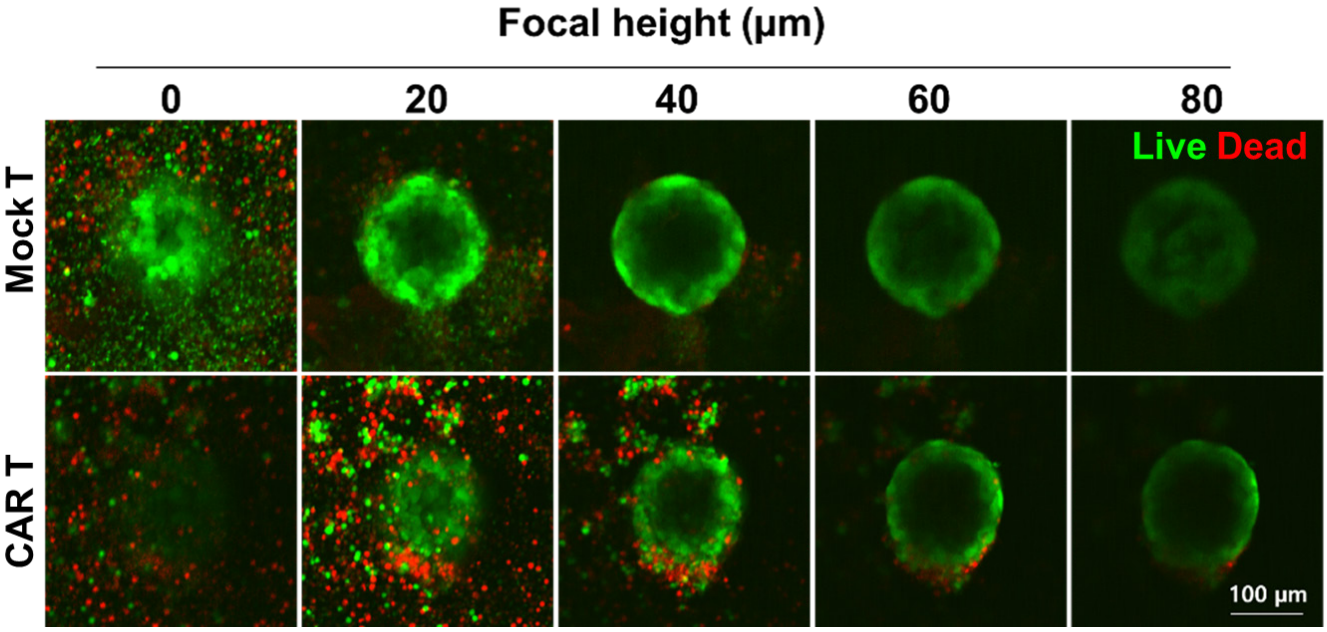

Figure S8. Confocal imaging of LIVE/DEAD stained BT474 spheroids treated with mock T or HER2 CAR T cells at a 1:4 ratio (500–2,000 cells) at 12 h captured by K1-Fluo confocal microscopy. Scale bar = 100 μm.

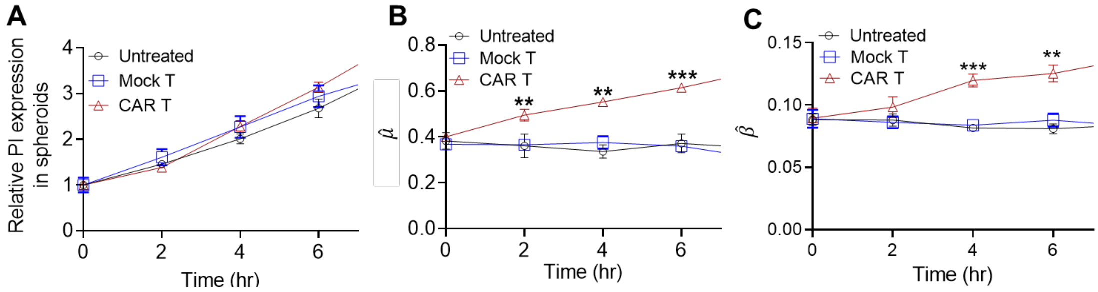

Figure S9. Time-dependent quantification of relative PI expressions in spheroids (A), $\hat{\mu}$ (B) and $\hat{\beta}$ (C) from 0 to 6 hrs. $\hat{\mu}$ and $\hat{\beta}$ could measure early stress responses or immune cell engagement compared to PI analysis. One-way ANOVA with Tukey's post hoc test: $**p < 0.01$; $***p < 0.001$. n = 3 for A; n = 9 for B and C.

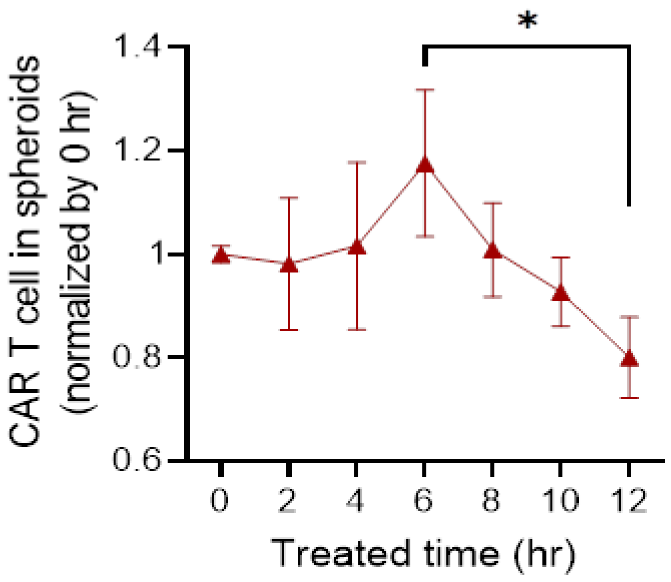

Figure S10. Quantification of CAR T cells in spheroids by measuring fluorescence of membrane labelled CAR T for 12 hr. The amount of CAR T didn't increase as the PI signal showing the CAR T exhibited the serial killing behavior. One-way ANOVA test followed by Tukey's post-hoc test. $p^* < 0.05$. Spheroid number = 3.

Reference

1. Kut C, Chaichana KL, Xi J, Raza SM, Ye X, McVeigh ER, et al. Detection of human brain cancer infiltration ex vivo and in vivo using quantitative optical coherence tomography. Sci Transl Med. 2015; 7: 292ra100.
2. Yuan W, Kut C, Liang W, Li X. Robust and fast characterization of OCT-based optical attenuation using a novel frequency-domain algorithm for brain cancer detection. Sci Rep. 2017; 7: 44909.
3. Vermeer KA, Mo J, Weda JJ, Lemij HG, de Boer JF. Depth-resolved model-based reconstruction of attenuation coefficients in optical coherence tomography. Biomed Opt Express. 2013; 5: 322-37.
4. Auslander L, Buffalano C, Orr RS, Tolimieri R. Comparison of the Gabor and short-time Fourier transforms for signal detection and feature extraction in noisy environments. Advanced Signal Processing Algorithms, Architectures, and Implementations: SPIE; 1990. p. 230-47.
